# miR-138-5p inhibits healing of femoral fracture osteogenesis in rats by modulating osteoblast differentiation via SIRT1/FOXO1 axis

**DOI:** 10.1186/s13018-025-05667-6

**Published:** 2025-03-14

**Authors:** Guangming Dai, Wei Jiang, Bo Feng, Lan Zhang

**Affiliations:** Department of Hand, Foot and Ankle Surgery, The Third Affiliated Hospital of Inner Mongolia Medical University, Inner Mongolia Autonomous Region, No.20, Shaoxian Road, Kundulun District, Baotou, 014010 P. R. China

**Keywords:** miRNA, miR-138-5p, SIRT1, FOXO1, Osteogenic differentiation

## Abstract

**Background:**

MicroRNAs have a crucial part to play in maintaining bone formation, signaling, and repair. This research explored the involvement miR-138-5p in modulating osteoblast differentiation in femoral fractures model.

**Methods:**

The role of mir-138-5p in the healing process of femoral fractures in rats was assessed through micro computed tomography (CT) imaging. After that, qPCR was employed to identify the cellular mRNA expression levels of miR-138-5p, SIRT1, and FoxO1 in either the callus or MC3T3-E1. Next, the protein expression level of Runx2, OPN, OCN and ALP was determined by western blot or ELISA. A dual-luciferase reporter gene assay was implemented to examine the target of miR-138-5p. The quantity of mineralized nodules was measured by means of alizarin red staining.

**Results:**

The miR-138-5p inhibitor promotes the mending of femoral fractures. When it is knocked down, the osteogenic differentiation is promoted, which may be caused by the enhanced activity of ALP and the elevation of the expression of Runx2, OPN and OCN. Meanwhile, an increase in the expression of mir-138-5p impairs the biosynthesis of SIRT1 and FoxO1. When SIRT1 and FoxO1 were downregulated with shRNA, the effect caused by the mir-138-5p inhibitor could be reversed.

**Conclusion:**

Our studies uncovered that the overexpressed miR-138-5p might have an inhibitory role in femoral fractures healing by inactivating SIRT1/FOXO1 axis.

## Highlight

We elucidated that miR-138-5p exerts a regulatory function through SIRT1/FOXO1 axis, providing new insights for the treatment of fracture healing by targeting miR-138-5p.

## Introduction

As growing in life expectancy, the incidences of femoral fractures in middle-aged and elderly people are on the rise [1]. Femoral fracture is defined as the destruction of the integrity or interruption of the continuity of the bone structure with the external force. Simultaneously, rupture of capillaries around the bone may lead to hematoma with edema in local tissues, which in turn affects blood circulation. If the swelling is not reduced in time, it may cause other complications such as post-fracture infection. Thus, further investigation on mechanisms of bone damage repair was necessary.

The bone healing requires the involvement of a variety of cells, including chondrocytes, mesenchymal stem cells, inflammatory cells, endothelial cells, osteoclasts, and osteoblasts [2]. For example, osteoblasts have been demonstrated to secrete a variety of extracellular matrix proteins, such as osteopontin, type I collagen, alkaline phosphatase, and osteocalcin, which play crucial roles in facilitating the differentiation of osteoblasts into osteocytes as they become embedded within the mineralized bone matrix [3]. Furthermore, osteoblasts were mainly differentiated from mesenchymal stem cells (MSCs) and skeletal stem cells (SSCs). In fact, fracture healing is essentially a process of bone resorption, bone formation, and bone remodeling and shaping that occurs at the broken end of a fracture. Osteoblasts primarily mediate bone formation, whereas osteoclasts primarily mediate bone resorption. By regulating the procedures of bone formation and clearance, it promoted the formation and proliferation of new bone tissue during the period of fracture healing, and facilitate the removal of callus, and ultimately completing the regeneration and remodeling of bone tissue.

The process of osteocytes maturation was modulated by a wide range of factors [4]. Initially, the expression of Sox9 was indispensable for the commitment of osteoprogenitor cell and inducing cell differentiation towards chondrocyte cell. Subsequently, the level of Runt-related transcription factor 2 (RUNX2) directed cell differentiation into preosteoblast. Finally, the expression of osterix and RUNX2 co-regulate the formation of mature osteoblasts. Similarly, macrophage colony-stimulating factor (M-CSF) had been indicated to promote proliferation of osteoclast precursors, whereas nuclear factor-κB ligand (RANKL) facilitated the differentiation of osteoclast precursors towards mature osteoclasts [5].

It has been reported that non-coding RNAs serve as crucial regulatory elements of bone formation, signaling, and repair, thus plays a pivotal role in bone-related disease [6–11]. For example, miR-138-5p has been shown to control the differentiation of aging osteoblasts through targeting MACF1 [12]. miR-138-5p promotes osteoclast apoptosis and inhibits osteoblast proliferation via targeting SIRT1 [13]. However, miR-138-5p has not been completely understood in femur fracture.

FOXO1 was recognized as a member of the FOXO transcription factor family and plays important roles in various biological processes, including cell proliferation, differentiation, metabolism, and stress response [14]. FOXO1 expression was up-regulated during bone formation [15]. Numerous studies have shown that FOXO1 promotes osteoblast differentiation and promotes osteogenesis by stimulating of the SIRT1/FOXO1 pathway, and it has been shown that the miR-138-5p/SIRT1/FOXO1 axis enhances wound healing [13, 16–17].

Above all, we proposed the hypothesis that miR-138-5p affects rat femur fracture healing by targeting SIRT1 to regulate the expression of FOXO1.

## Methods

### Rat closed femoral fractures model

A total of 36 male Sprague-Dawley rats were applied to construct a fracture model based on the fracturing the femoral diaphysis model methods reported by Bonnarens and Einhorn [18]. All animals prepared for surgery were fasted 8 h and intraperitoneal injection of 3% Pentobarbital sodium salt at 40 mpk. Once anaesthetized, the rats were positioned prone on the operating table, the surgical area of the right hind limb was shaved, sterilized with iodine povidone, and a sterile bore towel was laid. An incision was made over the medial knee. The medial patellar retinaculum was exposed and incised, extruding the patella, flexing the knee, exposing the femoral condyles. The inserting a 1.2-mm Kirschner needle into the femoral bone marrow cavity after the opening over the cruciate ligament, shortening the Kirschner needle, and pushing the tail end of the Kirschner needle into the femoral condyle. A 1.2 mm Kirschner pin was introduced into the canal following an incision was made above the cruciate ligament. The trailing end of the Kirschner pin was cut short and inserted into the femoral condyles. The wound was flushed with iodine-vodine saline, and the local incision was sutured. According to the Einhom fracture modelling method, a 500 g weight was dropped from a height of approximately 30 cm and impacted the mid femur, causing a short oblique fracture, which was further confirmed by micro-CT examination after completion. In addition, miR-138-5p-NC and miR-138-5p-inhibitor, which were synthesized by Genomeditech (Shanghai), were administered intravenously to animals. The animal experiment was authorized via the Animal Ethical and Welfare Committee of The Third Affiliated Hospital of Inner Mongolia Medical University (ID: 2019-MER-071).

### Micro computed tomography analysis of fracture callus formation

Sprague-Dawley rats (male, 8-week old) were divided into four group, including Sham, Model, Model + miR-138-5p-NC and Model + miR-138-5p-inhibitor. The healing of fractured femur subjected to different treatment for 8 weeks were evaluated by bone scanning using micro-CT instrument SMX-100CT (Shimadzu, Japan) [19–20]. The analysis of images was carried out using the CT analyzer program and the images were reconstructed in three dimensions. After 8 weeks of treatment, the local callus tissue at the fracture was dissected for further study.

### Cell culture

The MC3T3-E1 cell line were sourced from the Chinese Academy of Sciences located in shanghai, China. For resuspension, the MC3T3-E1 cell was treated with α-minimal essential medium (α-MEM, Procell, China) containing FBS (10%v/v, Beyotime, China) and 1% penicillin/streptomycin (10%v/v, Beyotime, China). These cells were put into a humidified incubator with a temperature of 37 °C and a CO₂ concentration of 5%. To induce osteogenic differentiation, the MC3T3-E1 cells were placed in the medium that having 10 mM Glycerol 2-phosphate (Medchemexpress, China) and 50 µg/mL ascorbic acid (Beyotime, China).

### Cell transfection

MC3T3-E1 cells were put onto 6-well plates and cultured in the cell incubator for 24 h during the logarithmic growth phase. Subsequently, the plasmids synthesized by Genscript (Nanjing, China), were transfected respectively by means of Lipofectamine^®^ 3000 reagent following the standard guidelines (Invitrogen, USA). The transfection efficiency was measured by conducting qPCR analysis 48 h after the transfection operation. The shRNA sequences utilized were as follows, from the 5’-end to the 3’-end [21–22]:

**Table:** 

Gene	Sequence
SIRT1	CCATTCTTCAAGTTTGCAA
FOXO1	CGCCAAACTCACTACACCATTTCAAGAGAATGGTGTAGTGAGTTTGGCTTTTTA

### Calcium nodule staining

MC3T3-E1 cells were inoculated into 24-well plates and subjected to stimulation under the corresponding conditions, and then cultured for 14 days to generate opaque calcified nodules. After discarding the medium, the wells were rinsed twice with PBS. Then, 500 µL of 4% paraformaldehyde was introduced into each well to fix the cells for 10 min. Following this, the wells were rinsed twice again with PBS. Subsequently, 0.4% alizarin red solution was applied for 20 min. After that, the cells were dried and photographed using an inverted light microscope after washing twice with PBS.

### qPCR

Total RNA of the callus tissue or MC3T3-E1 samples to be tested was extracted by means of Trizol reagent (Beyotime, China) following the manufacturer’s standard guidelines. Then, it was transcribed into cDNA employing the RevertAid First Strand cDNA Synthesis Kit (Thermo Fisher scientific, USA). The relative extent of target genes was determined by CFX Opus Systems (Bio-Rad, USA) with iTaq Universal SYBR Green Supermix kits (Bio-Rad, USA). U6 and GAPDH respectively functioned as an internal reference. The expression levels were analyzed employing the 2 − ΔΔCt methods. The primers sequences utilized were as follows, from the 5’-end to the 3’-end:

**Table:** 

Gene name	Primer sequence
miR-138-5p	Forward-CCCAGGGTCTGGTGCGGAGA
	Reverse- CAGGGGCTGAGCGGTGAGGG
SIRT1	Forward-F-GCGGCTCGTGTCACAGTCAG
	Reverse-TCCTCAAATGCAGCTTCCACTTCC
Foxo1	Forward-CGTCTCCTGGTACTCTCTGC
	Reverse-TGGACTGGTTAAACTCCGGC
GAPDH	Forward-AGCTTCGGCACATATTTCATCTG
	Reverse-CGTTCACTCCCATGACAAACA
U6	Forward-TTGACTCCACAAAAGGGAAGAAG
	Reverse-TCCAGAGGTCTGTTGAATCCG

### Dual-luciferase reporter gene assay

Initially, the Starbase website was utilized to forecast the likely binding site of SIRT1 and miR-138-5p [23]. Then, the wild and mutant type of SIRT1 promotor fragments were constructed into pmirGLO vector (Genomeditech, China) to produce SIRT1-MUT and SIRT1-WT reporter vectors, respectively. After that, MC3T3-E1 cells were co-transfected with SIRT1-MUT, SIRT1-WT as well as miR-138-5p mimics/inhibitor employing Lipofectamine 3000 reagent for 48 h. The design and synthesis of the wild-type (SIRT1-WT) and mutant (SIRT1-MUT) sequences were entrusted to Genomeditech. Specifically, bioinformatics tools were employed to predict the miR-138-5p binding site within the 3’ UTR of SIRT1. To generate the SIRT1-MUT construct, targeted mutations were introduced into the seed region of the predicted binding site, thereby disrupting the interaction with miR-138-5p. The luciferase activity analysis was executed by employing the Reporter Assay (E1910, Promega, USA).

### Western blot

The callus tissue sample and MC3T3-E1 cells that had been transfected with the corresponding plasmids were harvested and then broken down to obtain total protein. This was achieved by using RIPA Lysis Buffer (Beyotime, China) together with 10 µL PMSF while on ice. The determination of the total protein concentration was carried out by the BCA Protein Assay Kit (Solarbio, China). The same amount were separated by means of 10% SDS–PAGE (Genscript, China) and subsequently transferred to PVDF membranes (Beyotime, China). Then, QuickBlock™ Blocking Buffer (Beyotime, China) was utilized to block the blots for an hour. Subsequently, they were washed three times with TBST buffer (Beyotime, China). Thereafter, the incubation of primary antibodies was performed at 4 °C for 12 h. The specific antibodies and their details were: Anti-OCN (Bio-Techne, AF808, 1:2500), Anti-OPN (Proteintech, 22952-1-AP,1:3000), anti-RUNX2 (Proteintech, 20700-1-AP, 1:500), anti-SIRT1 (Proteintech, 13161-1-AP, 1:4000), anti-Foxo1 (Proteintech, 18592-1-AP, 1:3000), Anti-β-Actin (Proteintech, 66009-1-Ig, 1:20000). Afterwards, the blots were washed with TBST buffer and subjected to incubation with the HRP-linked secondary antibody (7074P2, Cell Signaling Technology) for an hour at 25 °C. The blots were visualized by treated with ECL reagents (12630P, Cell Signaling Technology) and then photographed applying the Amersham Imager 600 imagers (GE, USA). The obtained photos were assessed by Image J software.

### Alkaline phosphatase (ALP) activity assay

The microtiter plate was coated with an anti-ALP antibody to capture ALP from the serum of rats or culture medium of MC3T3-E1 (9018, Corning, USA). The ALP activity was examined at 400–415 nm absorbance employing an ALP activity kit (Beyotime, China) consulting the manufacturer’s specification.

### Statistical analysis

All of the results were presented in no fewer than three independent experiments and analyzed using GraphPad Prism 8 software. The values were given with the mean accompanied by the standard deviation, denoted as mean ± SD. The differences among different groups were assessed by One-way ANOVA. *P*-values under 0.05 were taken as significantly different.

## Results

### miR-138-5p inhibits osteogenic differentiation in femur fracture rat model

**Fig. 1:**
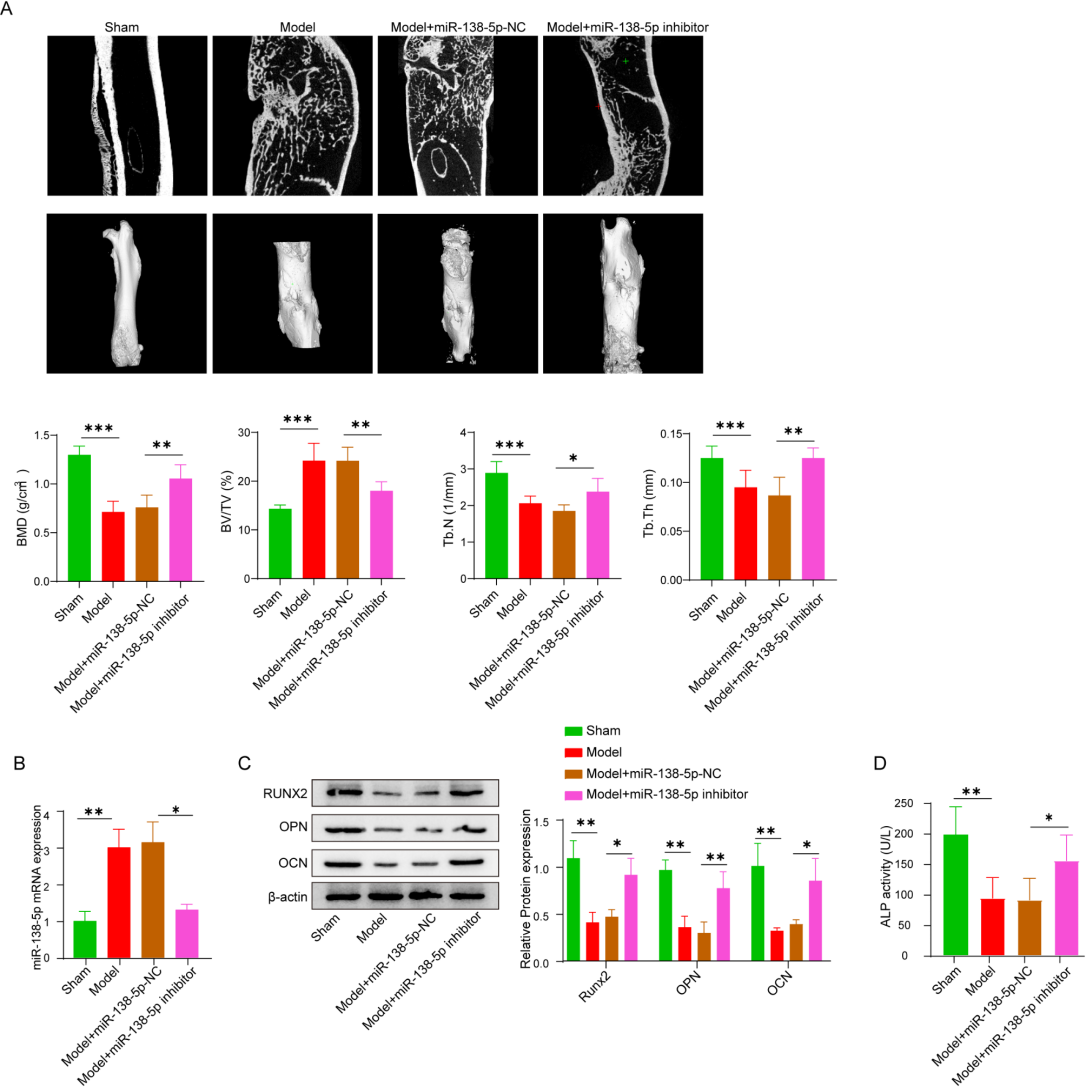
Effect of miR-138-5p on osteogenic differentiation in rats with femur fracture. (**A**) CT examination of osteogenesis, and bone morphometrical analysis of Bone Mineral Density (BMD), Bone volume/tissue volume (BV/TV), Trabecular thickness (Tb.Th) and Number of bone trabeculae (Tb.N). (**B**) The expression level of miR-138-5p in callus determined by qPCR. (**C**) The levels of Runx2, OPN, and OCN in the model group, miR-138-5p-NC group, sham and miR-138-5p-inhibitor group determined by western blot. (**D**) The plasma level of ALP in each group determined by ELISA. Data were displayed as mean ± SD. *p < 0.05, **p < 0.01, ***p < 0.001

For proof of concept, a femur fracture model was established in rats to explore the roles of miR-138-5p in fracture healing. The miR-138-5p-NC and miR-138-5p-inhibitor were intravenously administered into Sprague-Dawley rat, respectively. Obviously, these CT images showed that the fracture line was moderate larger in the model group by contrast with the Sham group. Upon the silencing of miR-138-5p through the administration of miR-138-5p inhibitor, there was a notable and statistically significant acceleration in the rate of bone healing (Fig. 1A). Furthermore, The micro-CT analysis revealed that, compared to the model group, the administration of the miR-138-5p inhibitor led to significant improvements in callus formation, bone mineral density (BMD), trabecular thickness (Tb.Th), and trabecular number (Tb.N) (Fig. 1A). Additionally, the bone volume fraction (BV/TV) was observed to decrease under these conditions (Fig. 1A). Subsequently, qRT-PCR was applied following manufacturer’s instruction to measure the expression level of miR-138-5p in osteoblasts [19]. Obviously, the cellular concentration was significantly elevated in the model group either with or without miR-138-5p NC (Fig. 1B). By contrast, a dramatical decreased in mRNA expression of miR-138-5p was observed upon addition of miR-138-5p inhibitor (Fig. 1B). Furthermore, western blot and ELISA were carried out to determine the level of osteoblast-related protein. It was discovered that the level of OCN, OPN, Runx2 and alkaline phosphatase were simultaneously attenuated in both the model and miR-138-5p-NC group (Fig. 1C and D). The treatment of miR-138-5p inhibitor would reverse this downward trend. These findings implied miR-138-5p affects fracture healing by inhibiting osteoblast differentiation in femur fracture rats.

### miR-138-5p inhibits osteogenic differentiation in vitro

**Fig. 2:**
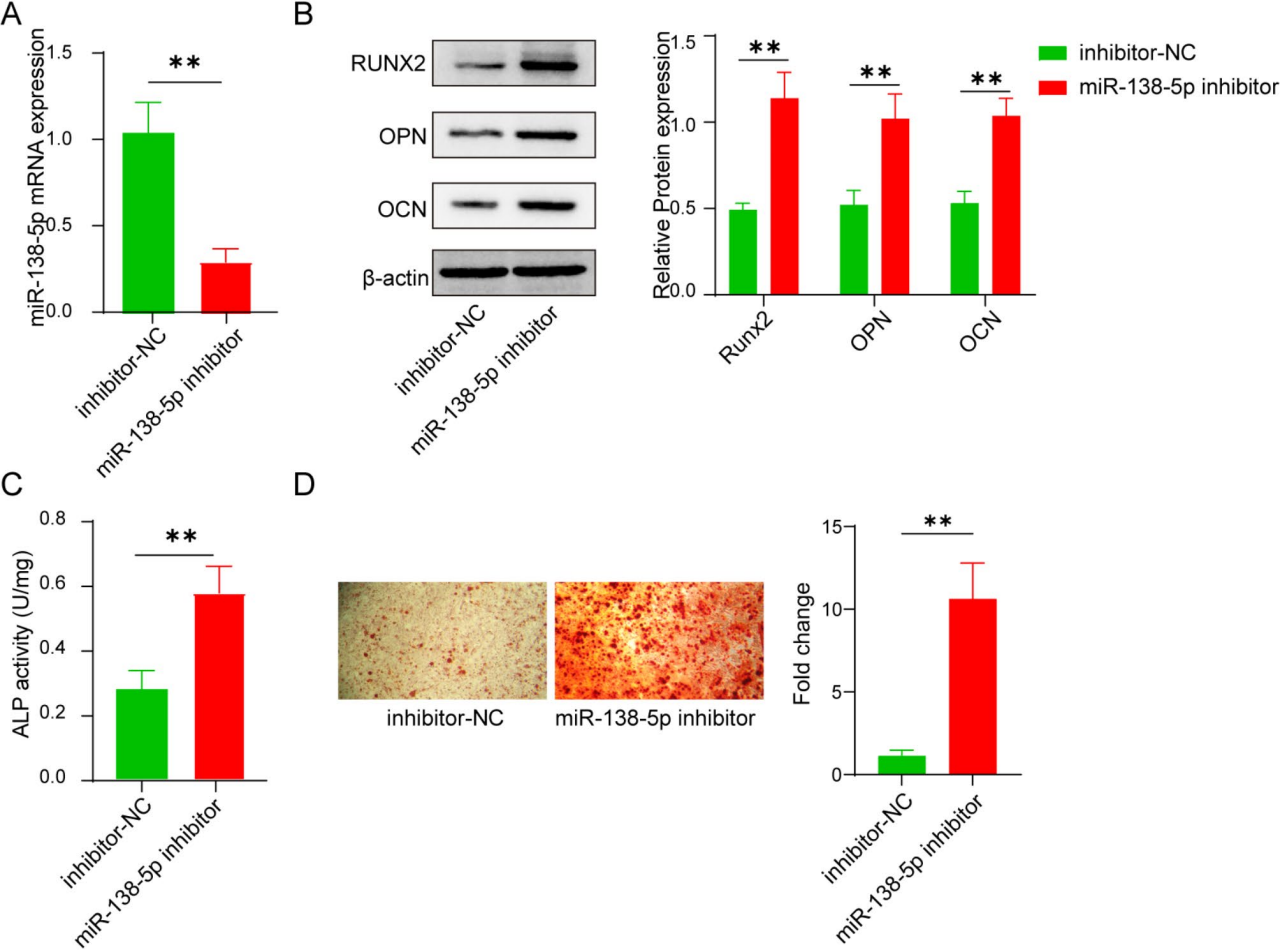
In vitro evaluation of miR-138-5p on osteogenic differentiation of MC3T3-E1 cells. (**A**) The cellular expression of miR-138-5p measured by qPCR. (**B**) The cellular levels of Runx2, OPN, and OCN with and without miR-138-5p inhibitor. (**C**) Evaluation of cellular ALP activity. (**D**) Measurement of the number of mineralized nodules with and without miR-138-5p inhibitor by Alizarin Red S Staining. Data were displayed as mean ± SD. *p < 0.05, **p < 0.01, ***p < 0.001

For the purpose of unveiling the regulatory roles of miR-138-5p in osteogenic differentiation, the MC3T3-E1 preosteoblast cells were cultivated for the purpose of observing diverse cellular activities. It was ascertained that the expression of miR-138-5p was substantially diminished within the miR-138-5p-inhibitor group (Fig. 2A). Moreover, the outcomes of western blot displayed that the protein expression level of OCN, OPN and RUNX2 were elevated subjected to the supplementation of miR‐138-5p inhibitor (Fig. 2B). Likewise, it was observed that the addition of miR‐138-5p inhibitor led to an increase in the activity of ALP (Fig. 2C). Finally, the negative control group was outnumbered by the mineralized nodules in miR-138-5p inhibitor group by three-folds (Fig. 2D). These results were in line with in in vivo results. However, more detailed mechanical studies need to be further investigation.

### miR-138-5p targets SIRT1 to regulate FOXO1 expression

**Fig. 3:**
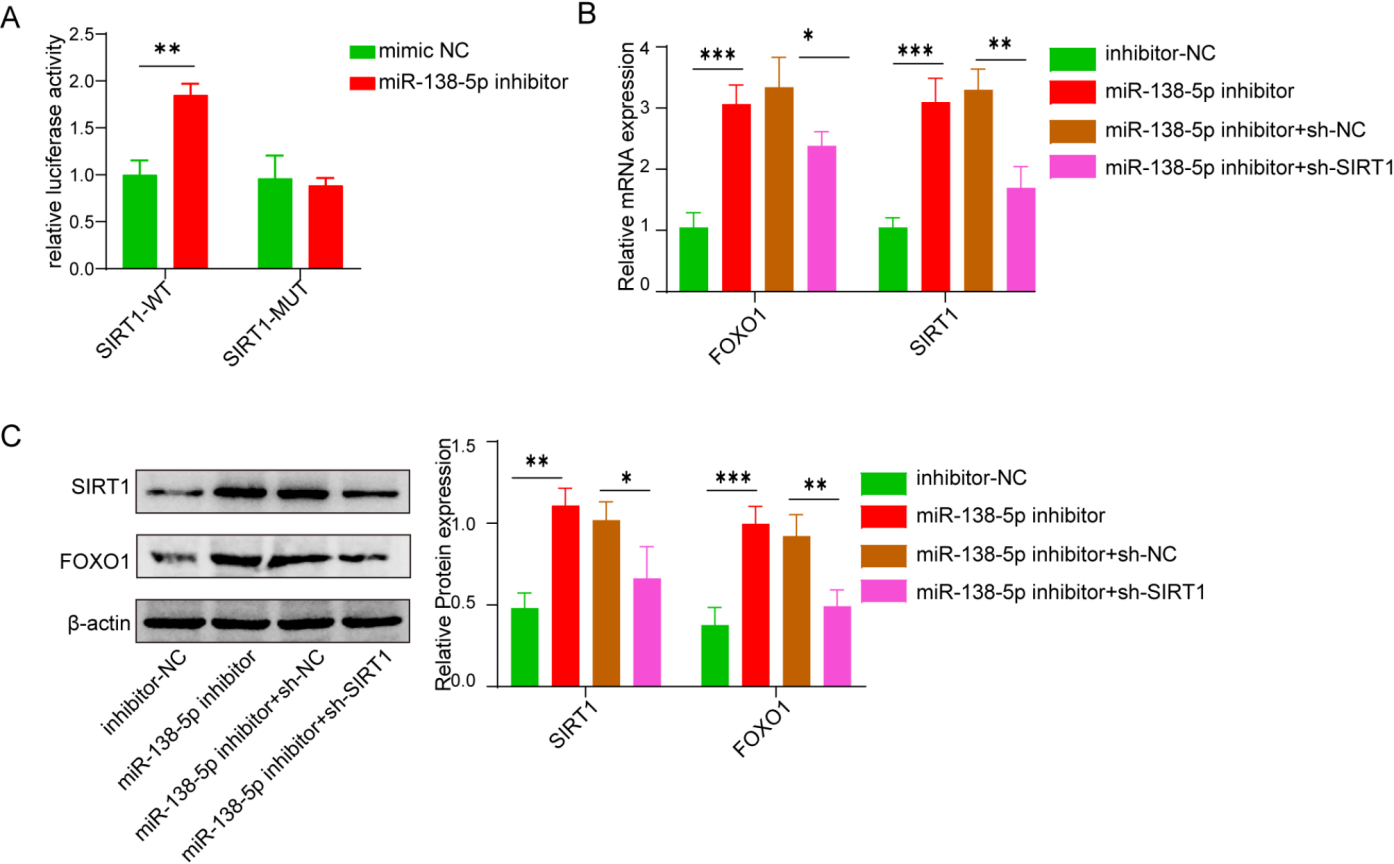
In vitro effect of miR-138-5p on SIRT1 and FOXO1 expression in MC3T3-E1 cells. (**A**) The luciferase activity change with and without miR-138-5p upon wild-type and mutated SIRT1. (**B**) mRNA level of SIRT1 and Foxo1 by qPCR in inhibitor-NC, miR-138-5p -inhibitor, miR-138-5p with sh-NC and miR-138-5p with sh-SIRT1 group, respectively. (**C**) The protein level of SIRT1 and Foxo1 by western blot in inhibitor-NC, miR-138-5p -inhibitor, miR-138-5p with sh-NC and miR-138-5p with sh-SIRT1, respectively. Data were displayed as mean ± SD. *p < 0.05, **p < 0.01, ***p < 0.001

Given that it has been reported that miR-138-5p may bind the UTR region of SIRT1 [17], we hypothesized that this interaction might modulate the stability of SIRT1 and further affect its downstream signaling pathways. Firstly, the impact of miR-138-5p on SIRT1 was examined through dual-luciferase reporter assay. In comparison with the inhibitor-NC group, the relative luciferase activity of SIRT1-WT was notably elevated subjected to the treatment of miR-138-5p inhibitor, while not remarkable in SIRT1-mutated system (Fig. 3A). This confirms that miR-138-5p was capable of binding to SIRT1 in vitro. Furthermore, the levels of SIRT1 and FOXO1 in inhibitor-NC and miR-138-5p-inhibitor were assessed. It was obvious that significantly higher levels were expressed by the latter than by the former. These results provide the insight that miR-138-5p might binding with SIRT1 to modulate FOXO1 expression in vitro. To further confirm, SIRT1 shRNA and scrambled shRNA were transfected into MC3T3-E1 cells subjected to the treatment of miR-138-5p inhibitor. Evidently, the up-regulation of SIRT1 mRNA expression that was induced by the miR-138-5p inhibitor was specifically counteracted by the addition of SIRT1 shRNA (Fig. 3B). Meanwhile, the FOXO1 expression was selectively decreased with the addition of SIRT1 shRNA upon pretreated using miR-138-5p inhibitor (Fig. 3B). Additionally, the presence of miR-138-5p inhibitor simultaneously up-regulated the cellular protein expression levels of SIRT1 and FOXO1. Once pretreated with miR-138-5p inhibitor, the expression of these protein would down-regulated (Fig. 3C). These results verified that miR-138-5p regulates expression by acting on SIRT1.

### miR-138-5p/SIRT1/FOXO1 axis inhibits osteogenic differentiation in vitro

**Fig. 4:**
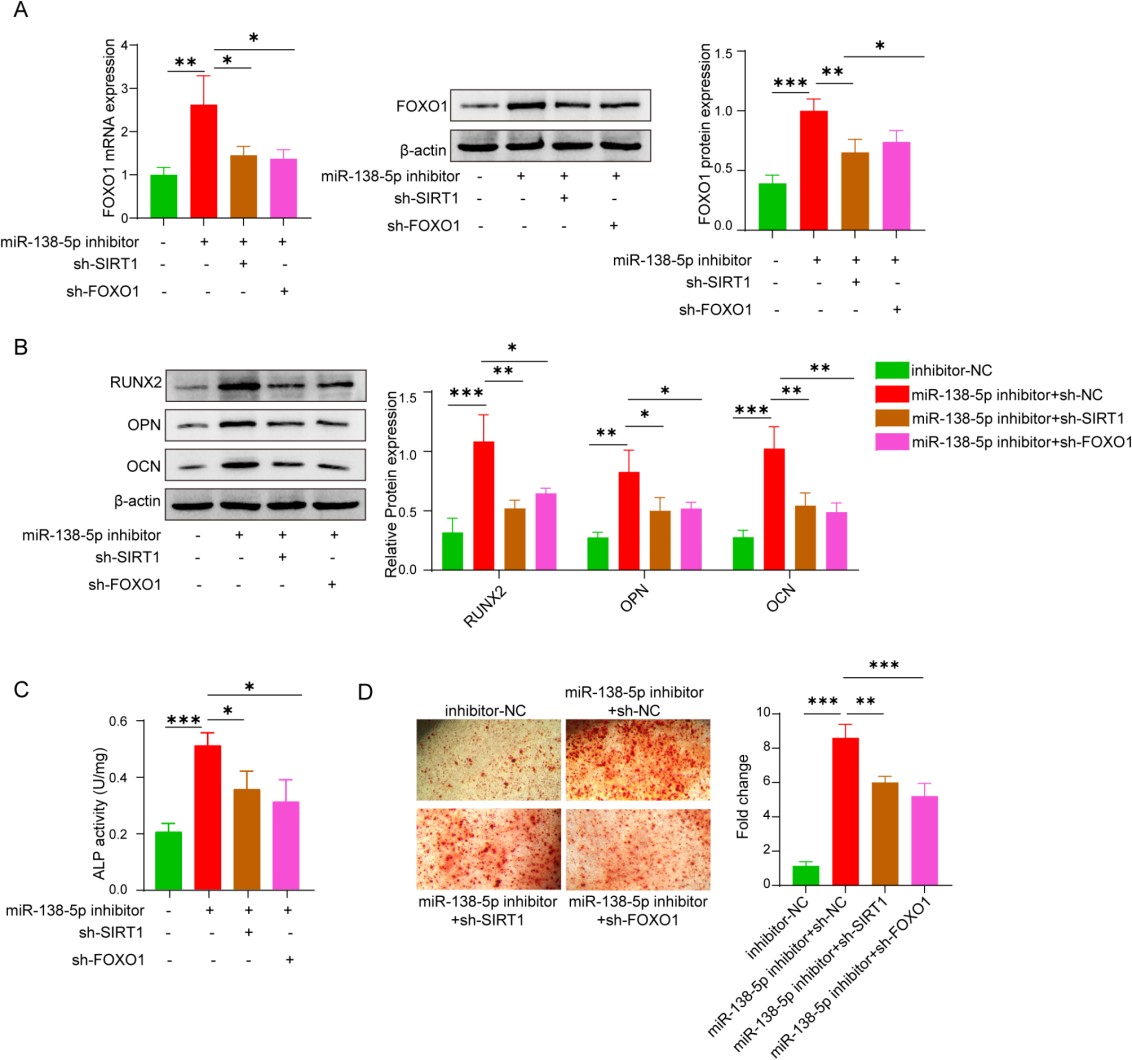
miR-138-5p inhibits osteogenic differentiation of MC3T3-E1 cells by SIRT1/FOXO1 axis. (**A**) FOXO1 mRNA and protein expression levels with and without miR-138-5p inhibitor, respectively. (**B**) The cellular levels of Runx2, OPN, and OCN with and without miR-138-5p inhibitor. (**C**) Evaluation of cellular ALP activity with and without miR-138-5p inhibitor. (**D**) Measurement of the number of mineralized nodules with and without miR-138-5p inhibitor by Alizarin Red S Staining. Data were displayed as mean ± SD. *p < 0.05, **p < 0.01, ***p < 0.001

miR-138-5p plays a substantial role in the differentiation process of osteoblasts in vivo as well as in vitro. Furthermore, it was demonstrated that mir-138-5p was capable of regulating the expression of FOXO1 via SIRT1. However, this doesn’t imply that miR-138-5p was governing osteoblast differentiation through this particular pathway, thus requiring further exploration. Obviously, it was found that the mRNA and protein level of FOXO1 was dramatically enhanced subjected to the supplementation of miR-138-5p inhibitor, whereas SIRT1 shRNA and FOXO1 shRNA would attenuate the expression of FOXO1 (Fig. 4A). Besides, it was suggested that the cellular protein level of OCN, OPN, RUNX2 and ALP were enhanced upon the treatment of miR-138-5p inhibitor (Fig. 4B and C). Regardless of whether shRNA SIRT1 or FOXO1 was added, the protein level of OCN, OPN, RUNX2 and ALP were simultaneously diminished (Fig. 4B and C). In addition, there were more mineralized nodules in the miR-138-5p inhibitor group than in the inhibitor-NC group. On the other hand, the number was diminished in the group using the miR-138-5p inhibitor together with shRNA SIRT1 and FOXO1 (Fig. 4D). Consequently, the aforementioned results were capable of verifying that miR-138-5p modulates FOXO expression by binding to SIRT1, and this ultimately has an impact on the differentiation of osteoblasts.

## Discussion

Femoral fractures, particularly in aging populations, pose significant clinical challenges due to delayed healing and complications such as non-union [24]. However, Fracture healing is a multifaceted biological process involving coordinated interactions among osteoblasts (OBs), osteoclasts, cytokines, and genetic regulators [25–26]. The present study demonstrates that miR-138-5p acts as a negative regulator of femoral fracture healing in rats by impairing osteoblast differentiation through suppression of the SIRT1/FOXO1 signaling axis. This finding provides critical mechanistic insights into the molecular pathways governing bone repair and highlights the therapeutic potential of targeting miR-138-5p to enhance fracture recovery.

miRNAs belong to a category of small single-stranded molecules that are capable of regulating mRNA translation and stability, thereby playing a pivotal role in the regulation of gene expression [27–28]. Available evidence to date reveals that miRNAs influence numerous diseases, namely cardiovascular disease, neurodegenerative disease, cancer, autoimmune disease, metabolic disease and bone-related diseases [6, 29–30]. For instance, miR-141 and miR-200a participated in the regulation of osteogenic differentiation by targeting Dlx5 within MC3T3-E1 cells [31]. Accumulating evidence has firmly established that miR-138-5p plays a crucial regulatory role in the differentiation of osteoblasts among the elderly population [12]. Consistently, our meticulously conducted animal experiments and in vitro assays have provided additional corroboration of this phenomenon. Indeed, when miR-138-5p was overexpressed, it caused a noticeably lower expression of cell differentiation biomarkers like Runx2, OPN, OCN, as well as a decline in the activity of ALP. These findings were in line with those of an earlier study concerning the impact of miR-138-5p in the process of osteogenic differentiation [13, 16]. These data demonstrated that miR-138-5p had the ability to restrain osteogenic differentiation both in cellular and animal level.

The available evidences was sufficiently to indicate that SIRT1 is the target of miR-138-5p [13, 17]. Indeed, it was uncovered that the attachment of miR-138-5p to SIRT1 within MC3T3-E1 cells by means of dual-luciferase reporter assay. Upregulated miR-138-5p was detrimental to SIRT1 gene expression, which in turn indirectly affects the expression of other genes. Through literature analysis, we found that exosomes promoted the healing of wound via the miR-138-5p/SIRT1/FOXO1 pathway and resveratrol improved osteogenesis by regulating SIRT1/ FOXO1 axis [16, 32]. In our study, we found that the trend of change in the expression level of FOXO1 was highly consistent with that of SIRT1. Knockdown of FOXO1 or SIRT1 counteracted the effects of downregulation of miR-138-5p to some content. Upon addition of shRNA SIRT1 or FOXO1, down-regulation of the expression of OCN, OPN, and Runx2, a decrease in the quantity of mineralized nodules and the ALP activity was observed. The experimental outcomes revealed that miR-138-5p/SIRT1/FOXO1 axis curbs the osteogenic differentiation process of MC3T3-E1 cells, further validating our proposed mechanism.

In our study, we discovered that miR-138-5p inhibits healing of femoral fracture osteogenesis in rats by modulating osteoblast differentiation via SIRT1/FOXO1 axis. However, this study has the following limitations: firstly, the experimental model is limited to the rodent animal, and the application of its research conclusions to human fracture healing still needs to be validated through clinical studies; Secondly, the interactive regulatory mechanism of miR-138-5p with inflammatory response and angiogenesis pathways during fracture repair has not been elucidated, and the timing and regulatory targets of this molecular network need to be further studied and analyzed.

## Conclusions

In the present work, we present the fact that miR-138-5p curbs osteoblast differentiation both in vivo and in vitro. More evidence from in vitro data suggests that the impact of miR-138-5p on the SIRT1/FOXO1 axis could be favorable to the restraint of osteoblast differentiation. This study raises the clinical possibilities for the treatment of diseases associated with fracture healing by targeting miR-138-5p. At the same time, the mechanism of mir-138-5p in regulating bone healing also needs to be further studied.

## Data Availability

The datasets used or analyzed during the current study are available from the corresponding author on reasonable request.

## Abbreviations

SIRT1: Silent information regulator factor 2-related enzyme 1
ALP: Alkaline phosphatase
qPCR: Quantitative real-time polymerase chain reaction
Runx2: Runt-related transcription factor 2
OCN: Osteocalcin
MSCs: Mesenchymal stem cells
MACF1: Microtubule actin crosslinking factor 1
FOXO1: Forkhead box protein O1
OPN: Osteopontin

## Declarations

### Generative AI and AI-assisted technologies in the writing process

During the preparation of this work the authors did not use any AI-assisted technology.
